# Conservative versus surgical treatment of 21 sports horses with osseous trauma in the proximal phalangeal sagittal groove diagnosed by low‐field MRI

**DOI:** 10.1111/vsu.12936

**Published:** 2018-09-14

**Authors:** Giulia Lipreri, Bruce M. Bladon, Maria Elisabetta Giorio, Ellen R. Singer

**Affiliations:** ^1^ Leahurst Equine Hospital, University of Liverpool Neston United Kingdom; ^2^ Donnington Grove Veterinary Group Newbury United Kingdom; ^3^ Institute of Chronic and Ageing Disease, University of Liverpool Liverpool United Kingdom

## Abstract

**Objective:**

To compare the outcome following conservative vs surgical management of sports horses with a diagnosis of subchondral bone trauma of the proximal aspect of the proximal phalanx (PP) by low‐field MRI.

**Study design:**

Retrospective case series.

**Animals:**

Twenty‐one mature sports horses with evidence of high water signal within the proximal sagittal groove of the PP according to low‐field MRI of the fetlock, with no definitive radiographic evidence of fracture.

**Methods:**

Medical records (2010‐2017) of horses admitted to 2 referral hospitals were reviewed. Historical, clinical, radiographic, and MRI findings and treatment choice were recorded. Conservative management consisted of confinement to a stall followed by gradual reintroduction to exercise. Surgical management consisted of cortical screw fixation across the proximal aspect of the PP. Long‐term outcome was determined by telephone questionnaire or by clinical records review. Fisher's exact test was used to compare outcome between the 2 treatment groups.

**Results:**

Follow‐up was available in 17 of 21 horses in the study, including 8 horses treated conservatively and 9 treated surgically. No difference in outcome was detected in this study; 4 of 8 horses were sound after conservative management, and 5 of 9 horses were sound after surgery (*P* > .99).

**Conclusion:**

Approximately half of the horses that had a diagnosis of osseous trauma within the proximal aspect of PP returned to athletic activity, regardless of conservative or surgical management.

**Clinical significance:**

The prognosis for return to athletic functions in horses with osseous trauma within the proximal aspect of the PP is guarded.

## 1 INTRODUCTION

Osseous trauma within the subchondral bone and the adjacent trabecular bone of the proximal sagittal groove of the proximal phalanx (PP) is now recognized as a cause of lameness in thoroughbred racehorses and sports horses.^1^, ^2^ Osseous lesions within the sagittal groove could represent incomplete sagittal fractures of the PP, osseous cyst‐like lesions, or subtle subchondral bone changes.^1^, ^2^, ^3^, ^4^ Overt incomplete PP fractures are usually diagnosed radiographically; however, MRI often reveals subchondral bone abnormalities that would not be detectable with radiography.^1^ Subchondral bone injuries present a typical signal pattern on MR images that is characterized by T2‐weighted and short τ inversion recovery (STIR) sequence hyperintensity with corresponding T1‐weighted hypointensity.^3^ Typically, the hyperintense signal is associated with an enclosing hypointense halo, a fluid‐fat phase cancellation artefact on T2* gradient echo (GRE) out‐of‐phase sequences. The bone abnormalities result in a water‐based signal on MR images, which will be referred to as *high water signal* in this study. There is no clear evidence to indicate whether this typical water signal pattern is indicative of an acute traumatic event or of a more chronic process. The findings of Gold et al^4^ and Dyson et al^1^ support the hypothesis that osseous injury to the proximal sagittal groove of the proximal phalanx results from chronic repetitive trauma rather than from a single traumatic event.^1^, ^4^ A related theory would propose that osseous injuries of the proximal sagittal groove represent a spectrum of disease that can manifest as either an acute or a chronic clinical lameness.

Brunisholz et al^5^ described computed tomography (CT) conformation of incomplete proximal fractures of the PP in 24 sports horses.^5^ The fractures were located mainly in the sagittal plane, originating in the articular surface of the proximal sagittal groove of the PP. The presence of subchondral bone sclerosis, periosteal new bone formation, osteoarthritic abnormalities of the metacarpo/tarsophalangeal joint, and moderate degree and long duration of the lameness were frequently reported.^5^ By using different imaging modalities such as radiography, CT, and MRI, it is likely that various studies have described the same or related pathology.^1^, ^4^, ^5^ The pathogenesis of osseous trauma in the sagittal groove of PP may result from a combination of acute and chronic trauma, which would account for the variable appearance on different imaging modalities.

The causes of pain in diseased subchondral bone are currently poorly defined. Potential contributors to pain include raised intraosseous pressure; hypertension; invasion of sensitive structures such as the periosteum, ligaments, or joints by the structural damage; and the bone remodeling cascade.^6^ Because the pathogenesis of subchondral bone disease in the proximal sagittal groove of PP is not completely understood, the selection of the most appropriate treatment strategy is difficult, particularly when only MRI abnormalities are detected with unremarkable radiographic findings. Sports horses with radiographic evidence of short incomplete sagittal plane fractures of the PP had a more favorable clinical and radiographic outcome when treated by internal fixation compared with those treated conservatively.^7^ However, there is no clear evidence to determine the best treatment option for horses with a diagnosis of a short incomplete fracture of PP or evidence of subchondral bone injury according to low‐field MRI, with no or only subtle radiographic abnormalities. Currently, conservative treatment is the most common and consists of modifying the type and intensity of the exercise.^1^, ^2^, ^4^ Alternative treatments consist of either placing a cortical screw by using standard lag technique or forage of the proximal aspect of PP in the region of the lesion.^8^ The rational for this treatment is based on the recommendation for horses with dorsal cortical fractures in MCIII, in which osteostixis is performed.^9^

The objective of this study was to compare the outcome following conservative vs surgical management of subchondral bone trauma of the sagittal groove of the PP in sports horses diagnosed by MRI. Our hypothesis was that placement of an AO 4.5‐mm cortical screw in lag fashion across the area with high water signal would increase the proportion of horses achieving long‐term soundness compared with those receiving only conservative management.

## 2 MATERIALS AND METHODS

### 2.1 Inclusion criteria and study design

Clinical records of sports horses that had undergone MRI examination of the fetlock region between January 2010 and March 2017 were reviewed. Horses included in the study were initially presented for lameness evaluation, and the pain causing lameness was localized to the distal aspect of the limb by clinical examination, diagnostic analgesia, or scintigraphic examination. Absence of obvious radiographic findings warranted MRI examination. A number of the horses included in the study had foot MRI initially; however, with no obvious abnormalities detected by foot MRI, the decision was made to perform fetlock MRI. Horses with MR images revealing evidence of high water signal within the subchondral bone of the sagittal groove of the proximal phalanx and no or subtle radiographic abnormalities in the region of interest were included in the study. Horses in training for racing, horses with definitive radiographic evidence of fracture of the proximal phalanx, and horses with other concomitant orthopedic lesions likely to be the primary cause of lameness were excluded from the study.

### 2.2 Clinical features

Data obtained from medical records included breed, age, sex, use, level of competition, and affected limb. The level of competition was subjectively classified as *high* for international competitions, *intermediate* for British affiliated competitions, and *low* for British unaffiliated competitions. Lameness variables that were recorded included degree of lameness on presentation (Wynn‐Jones 1‐10/10 scale),^10^ nature of onset (acute/chronic), and duration. Lameness was considered acute if there was a sudden onset with no previous history of lameness and was considered chronic if there was a subtle onset or history of intermittent lameness that progressively worsened over several weeks before presentation. Lameness examinations were performed either by first opinion equine‐only veterinary surgeons or by European College of Veterinary Surgeons (ECVS) Diplomats working at the referral hospitals where MRI was performed. The results of diagnostic tests were reviewed, including diagnostic analgesia, radiography, and scintigraphy, if performed.

### 2.3 Radiographic features

Radiographic images of the metacarpo/metatarsophalangeal joint were were evaluated retrospectively, unblinded by the first author (GL). Lateromedial, dorsolateral‐palmaro (plantaro) medial, dorsomedial‐palmaro (plantaro) lateral, and dorsal 10° proximal‐palmarodistal (or dorsal 15° proximal‐plantarodistal) oblique radiographs were available for all horses. Presence of irregular periosteal and/or endosteal new bone in the dorsoproximal quadrant of the proximal phalanx, ill‐defined areas of radiolucency within the sagittal groove of PP, and osteoarthritic changes of the metacarpo/metatarso‐phalangeal joint were recorded.

### 2.4 MRI features

MRI of the fetlock region was performed with a low‐field (0.27 Tesla) permanent magnet (Hallmarq Veterinary Imaging, Guildford, United Kingdom) with the horse standing and sedated. T1‐weighted GRE, T2*‐weighted GRE, T2‐weighted fast spin echo, and STIR sequences were acquired in transverse, dorsal, and sagittal planes. Sagittal images were produced by orienting slices parallel to the lateral and medial aspect of the PP.^11^ Frontal images were produced by orienting slices parallel with the dorsal and palmar/plantar aspect of the proximal phalanx.^11^ Transverse images were produced by orienting slices perpendicular to the longitudinal axis of the proximal phalanx.^11^ MR images were reviewed by a single observer (GL), who was blinded to the patient treatment. Data recorded from MRI included presence of high water signal within the proximal sagittal groove of the PP, distribution of the high water signal within the sagittal groove (dorsal, middle, palmar/plantar aspect, or entire length of the sagittal groove), and presence of a fracture line within the area of high water signal.

### 2.5 Treatment

Treatment of choice was either conservative or surgical, depending on owner or surgeon preference. Conservatively managed horses were confined to a stall for 4‐6 weeks, followed by 1 month of in‐hand walking exercise, followed by a period of paddock turnout (3‐6 months). Surgically managed horses underwent placement of an AO 4.5‐mm cortical bone screw inserted in lag fashion across the PP under radiographic guidance, either under general anesthesia or under standing sedation and local anesthesia. Surgery was performed by ECVS board‐certified veterinary surgeons. The screw was placed in conventional lag fashion by using radiographic guidance. A 4.5‐mm glide hole was created, extending just beyond the sagittal midline. A centering sleeve was placed, after which a 3.2‐mm pilot hole was drilled through the transcortex. The hole was then countersunk, measured, and tapped (if indicated), and a 4.5‐mm cortical screw was inserted and tightened. In standing cases, a self‐tapping screw was used. The screw was positioned as proximally as possible while remaining distal to the articular surface of the sagittal groove of the PP. The technique that was used was derived from the technique for repair of sagittal fractures of the PP.^12^ If the abnormal MRI signal was distributed along the entire length of the sagittal groove without evidence of a fracture, the screw was placed halfway between the dorsal and palmar/plantar aspect of the PP. If the high water signal was more localized, the screw position was then adjusted to pass through the region of high water signal. After surgery, patients were kept on stable rest for 4 weeks and then gradually reintroduced to exercise and turn‐out in a similar manner to the conservatively managed patients.

### 2.6 Follow‐up

A telephone questionnaire (see Supporting Information) or review of clinical records was used to establish present level of soundness and exercise of the horses included in the study. Published competition results were also reviewed for horses competing at affiliated level. On the basis of the time between commencement of the treatment and the telephone questionnaire, the follow‐up was classified as perioperative (≤3 months), short‐term (>3‐6 months), medium‐term (>6‐12 months), or long‐term (>12 months).^13^ Only horses with long‐term follow‐up were included. The outcome was reported as presence/absence of lameness (according to owner response or follow‐up data available in clinical records) and return to lower, previous, or higher level of exercise compared to before the condition was diagnosed. Occurrence of complications was also recorded. Complications were subjectively classified as *catastrophic*, *major*, or *minor* according to the definitions and criteria reported by Cook et al.^13^

### 2.7 Statistical analysis

Fisher's exact test was used to compare outcome between the 2 treatment groups as well as the effect of limb distribution (hind limb vs forelimb), onset of lameness (acute vs chronic), and presence of radiographic abnormalities. To analyze the effect of degree of lameness, level of exercise/competition, and distribution of the water signal, 2 descriptive categories were combined (mild and moderate vs severe lameness, high and intermediate vs low level of exercise, focal vs dorsal‐to‐palmar/plantar distribution of high water signal across the sagittal groove).

## 3 RESULTS

### 3.1 Signalment

Twenty‐one horses met the inclusion criteria: 18 warmblood, 1 thoroughbred cross, 1 Andalusian, and 1 Arabian. Median age was 10 years (range, 5‐13). There were 8 mares, 12 geldings, and 1 stallion. Nine horses were competing in dressage, 7 in eventing, and 2 in show jumping. Two horses were used for general riding and 1 for showing. Ten horses were competing at high level, 4 at intermediate level, and 5 at low level; level of competition was unknown in 2 cases.

### 3.2 History and clinical examination

Duration of the lameness ranged between 0 and 73 days (median, 30); onset was described as acute in 14 cases and chronic in 3 cases. Information regarding onset was not available in 4 cases. A front limb was affected in 15 cases, and a hind limb was affected in 6 cases. Degree of lameness ranged between 0 and 8 of 10 (median, 3/10). The pain causing lameness was localized to the distal limb/fetlock region by diagnostic analgesia in 18 cases, by scintigraphic examination in 1 case, and by clinical examination in 2 cases. Lameness improved noticeably following palmar nerve blocks performed at the base of the proximal sesamoid bones in 11 of 18 cases, following palmar/plantar digital nerve block performed just proximal to the cartilages of the foot in 2 of 18 cases, and following low 4‐point nerve block (perineural analgesia of the palmar/plantar nerves performed at the junction of the proximal three‐quarters and the distal one‐quarter of the metacarpal/metatarsal region and of the palmar/plantar metacarpal/metatarsal nerves performed at the distal end of the second and fourth metacarpal/metatarsal bones) in 2 of 18 cases. In 6 horses, intra‐articular anesthesia of the fetlock joint was also performed, and the lameness was abolished in 3 of the 6 cases.

### 3.3 Radiographic findings

**Figure 1:**
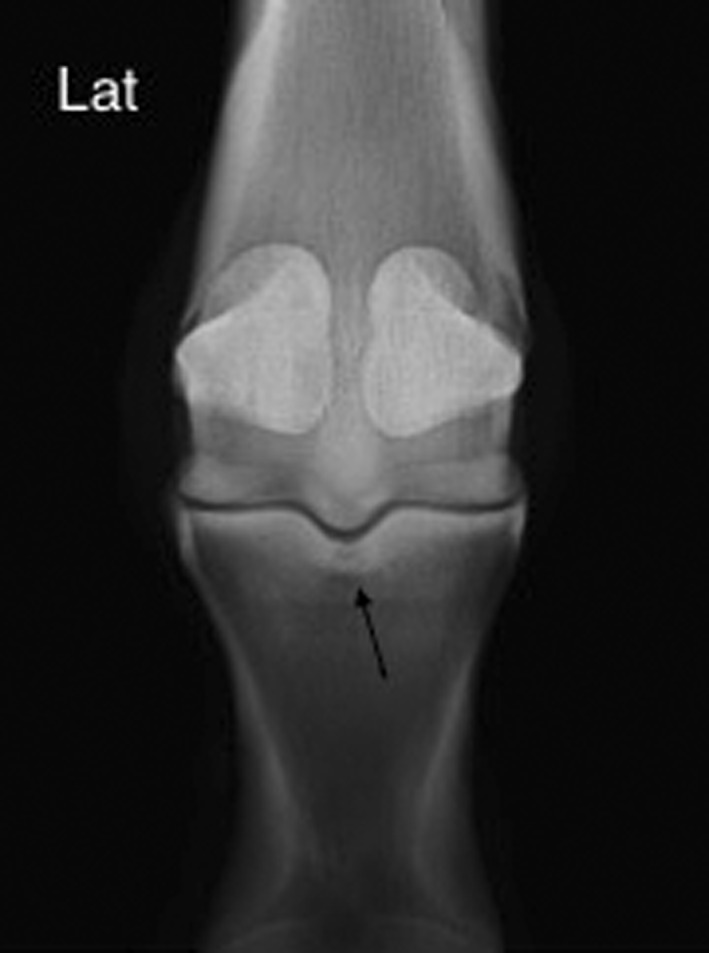
Dorsopalmar view of the metacarpophalangeal joint of a horse diagnosed with osseous trauma of the sagittal groove of the proximal phalanx. Medial is to the right. There is an ill‐defined radiolucent region surrounded by a more radiopaque area within the subchondral bone of the proximal phalangeal sagittal groove (arrow)

Radiological results revealed irregular periosteal new bone in the dorsoproximal quadrant of the PP in 5 horses and an ill‐defined area of radiolucency within the sagittal groove of the PP in 5 horses (Figure 1).

### 3.4 MRI findings

**Figure 2:**
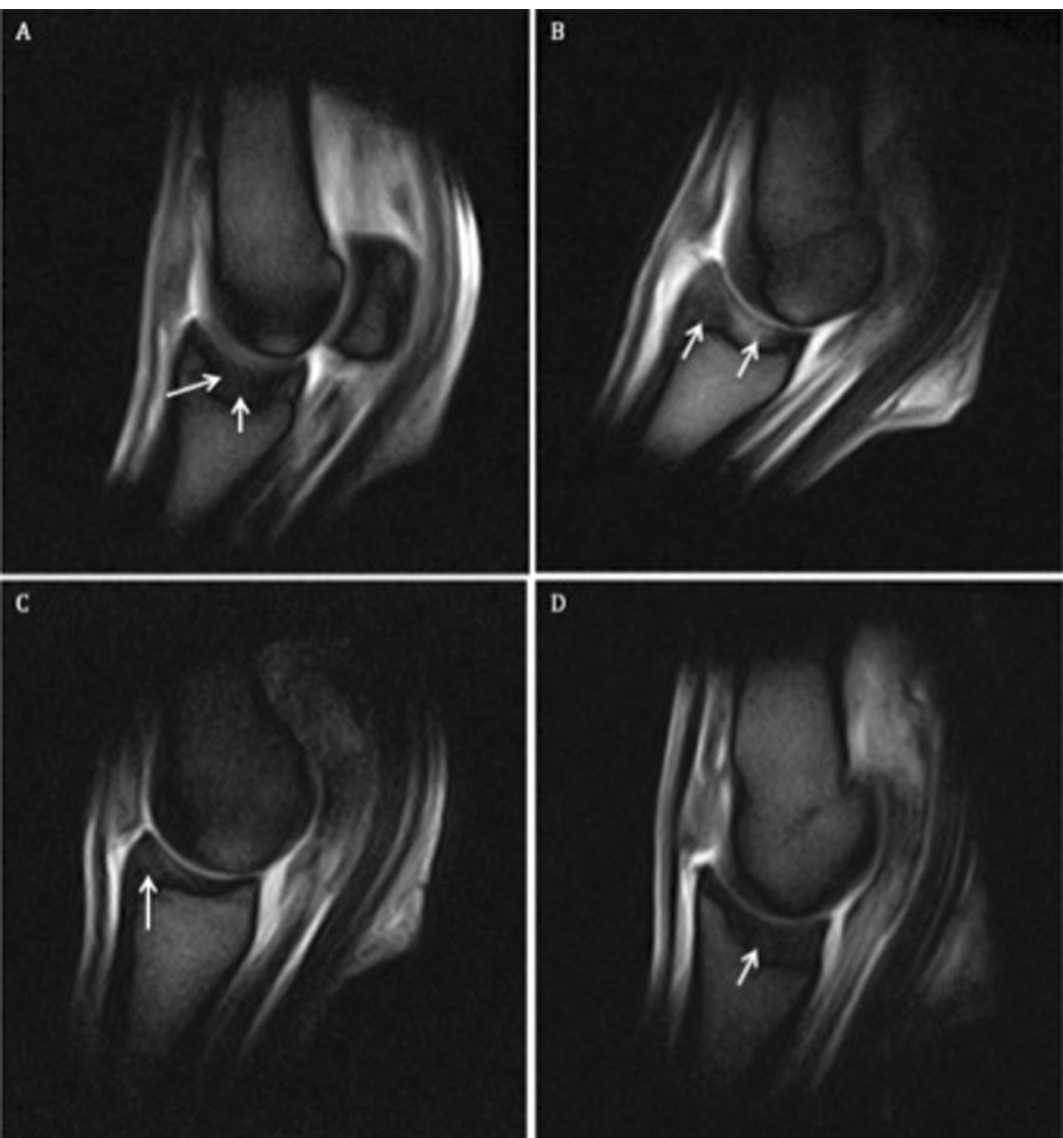
Sagittal T2*weighted gradient echo images of the metacarpo/metatarsophalangeal joint of horses with osseous trauma of the sagittal groove of the proximal phalanx. **A,** Area of high signal intensity (arrows) in the central region of the sagittal groove of PP affecting the subchondral and trabecular bone. **B,** Area of high signal intensity (arrows) extending from the dorsal to the palmar aspect of the sagittal groove of PP affecting the subchondral and trabecular bone. **C,** Area of high signal intensity (arrow) in the dorsal half of the sagittal groove of PP affecting the subchondral and trabecular bone. **D,** Area of high signal intensity (arrow) in the plantar half of the sagittal groove of PP affecting the subchondral and trabecular bone

**Figure 3:**
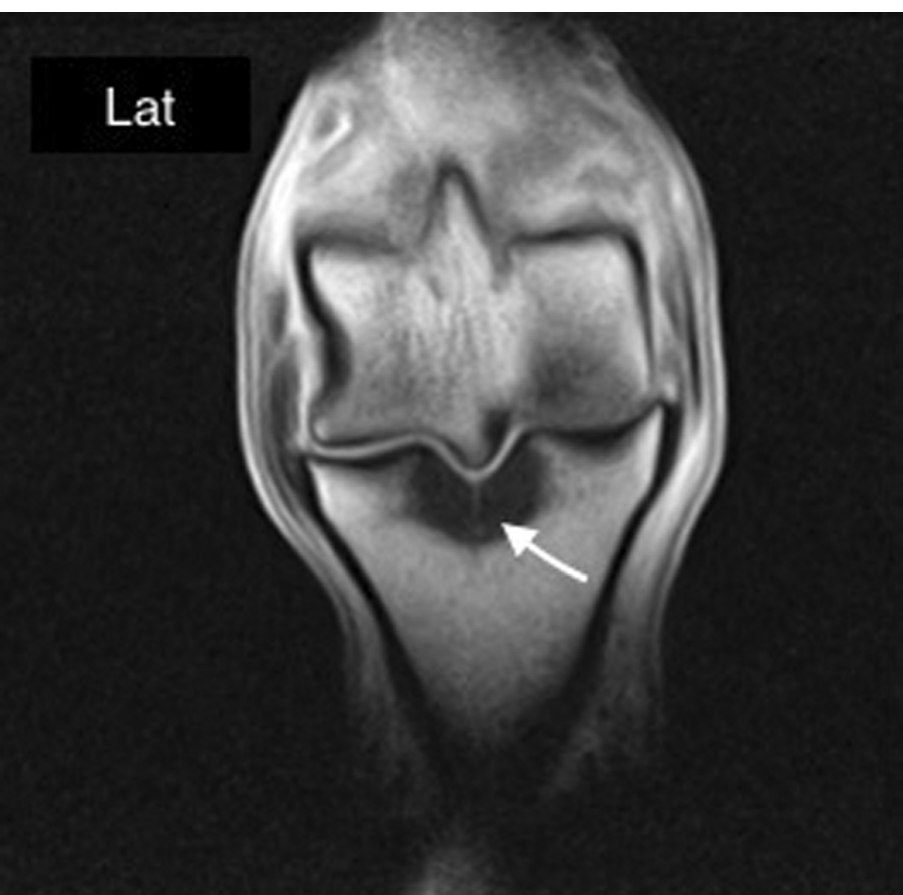
Dorsal T1‐weighted gradient echo image of the metatarsophalangeal joint. Medial is to the left. There is a linear high‐intensity signal (arrow) consistent with a fracture line within the sagittal groove extending from the articular surface through the subchondral bone and the trabecular bone of the proximal phalanx. An area of low signal intensity surrounds the fracture line

MRI revealed evidence of high water signal in all 21 horses included in the study. In 10 horses, the abnormal signal was localized in the central region of the sagittal groove, approximately halfway between the dorsal and palmar/plantar aspect of proximal aspect of the PP (Figure 2A). In 8 horses, the abnormal signal extended from the dorsal to the palmar/plantar aspect of the sagittal groove (Figure 2B). In 2 cases, the high water signal was localized in the dorsal half of the sagittal groove (Figure 2C), and in only 1 case it was localized in the plantar half (Figure 2D). Among the 5 horses that had radiographic evidence of periosteal new bone formation on the dorsoproximal aspect of the PP, 1 had high water signal localized in the dorsal aspect of the sagittal groove, 1 had high water signal localized in the central region, and 3 had high water signal extending from the dorsal to palmar aspect of the sagittal groove. An incomplete mid‐sagittal fracture line was evident within the high water signal in 6 horses (Figure 3). The fracture line was characterized by a linear high signal intensity in all sequences extending through the subchondral bone and the trabecular bone of the proximal phalanx. In 1 case, the plantar cortex was involved. Four of the 6 identified fracture lines (2 hind limbs and 2 forelimbs) were located in the central region of the sagittal groove, midway between the dorsal and palmar/plantar aspect. In 1 hind limb case, the fracture was located in the more plantar third of the proximal articular surface of PP, and in 1 forelimb case the fracture started in the most dorsal third of the articular surface. Only 2 of 6 cases in which a fracture line was identified by MRI had periosteal new bone formation on the dorsoproximal aspect of the PP according to radiographic examination. The fracture line was located in the most dorsal third of the sagittal groove in 1 case and in the central region in the second. In both cases, the high water signal extended from the dorsal to palmar aspect of the sagittal groove.

### 3.5 Treatment

Twelve horses were managed conservatively, and 9 were treated surgically. Nine of the horses managed conservatively had a forelimb affected, and 3 had a hind limb affected. Within the group treated surgically, 6 forelimbs and 3 hind limbs were operated on. The surgical procedure was performed under standing sedation and local anesthesia in 7 cases and under general anesthesia in 2 cases. No short‐term complications were recorded following surgery. In 1 horse, the screw was removed 18 months after surgery because of painful reaction on palpation of the screw head (minor complication). In 3 of the 9 horses that were treated surgically, the initial treatment was conservative. In 1 of these 3 cases, the surgical procedure was performed 8 months after the initial magnetic resonance examination because of persistence of the lameness. In the other 2 cases, the decision to perform surgery was made when lameness reoccurred after introduction of exercise, approximately 3 months after the initial MRI. Three of the 6 cases in which a fracture line was detected within the high water signal were treated surgically in the first instance, and 1 was initially managed conservatively. In the other 2 cases, conservative treatment was chosen.

### 3.6 Follow‐up and outcome

Long‐term follow‐up was available for 17 of the 21 horses and ranged from 12 to 36 months after diagnosis. Long‐term follow‐up was available for 8 horses that had been treated conservatively and 9 horses that had been treated surgically. Conservative management resulted in 4 of 8 horses becoming sound, 2 performing at the same level as before the injury, and 2 performing at a higher level. Surgical management resulted in 5 of 9 horses becoming sound, 4 performing at the same level as before the injury, and 1 performing at a higher level. Among the 4 horses that were still lame after surgery, 1 had surgery as the first treatment option, and 3 had surgery after failed conservative management. According to Fisher's exact test, there was no significant difference in outcome between conservative and surgical management (*P* > .99). When surgical and conservative treatment groups were combined, 9 of the 17 horses were sound at the time of the follow‐up. Among the 6 cases with diagnosis of an incomplete fracture, the 3 that were treated surgically in the first instance were all sound at the time of the follow‐up, and the 1 that had a delayed surgical intervention had a poor outcome. Unfortunately follow‐up was not available for the other 2 cases managed conservatively.

Were the 3 cases that had a delayed surgical intervention excluded, the outcome would be more favorable for surgically treated horses, with 1 of 6 horses being lame and 5 of 6 horses sound at the time of follow‐up. However, comparison with Fisher's exact test indicated that the difference in these proportions was still quite likely by chance alone (*P* = 0.3).

The effect of variables other than treatment choice on outcome was also investigated with Fisher's exact test. It was found that limb distribution, regardless of the treatment chosen, did not significantly affect outcome (*P* = 0.3). Among those horses that had a forelimb affected, 5 were treated conservatively, and 6 were treated surgically. Three of 5 horses that had been treated conservatively and 2 of 6 that had been treated surgically were sound at the time of follow‐up. On the other hand, 3 of the horses that had a hind limb affected were managed conservatively, and 3 were managed surgically. All the surgical candidates were sound at the time of follow‐up, and 2 of 3 horses that had been managed conservatively were sound.

Effect of acute compared to chronic onset of lameness on outcome was investigated. Eight of the 11 horses that had an acute onset of lameness were sound at follow‐up, and 1 of 3 that had a chronic lameness before presentation was sound. No statistical association between type of lameness onset and outcome was found (*P* = 0.5). The presence or absence of subtle radiographic changes did not significantly affect outcome (*P* = 0.6), with 4 of 8 horses with radiographic changes and 6 of 8 horses with no radiographic changes being sound at the time of follow‐up. Degree of lameness (*P* = 0.5), level of exercise/competition (*P* = 0.1), and distribution of the high water signal across the sagittal groove (*P* = 1) were not found to have statistically significant associations with outcome.

## 4 DISCUSSION

The results of this study provide no evidence of significant difference in outcome for horses with evidence of osseous trauma to the sagittal groove of the PP diagnosed by MRI when comparing conservative vs surgical treatment. In both treatment groups, approximately half of the cases had a favorable long‐term outcome. The MRI findings reveal a spectrum of disease from subchondral bone trauma to fracture within the sagittal groove, which may account for the variable response to treatment.

Our overall outcome is similar to the report by Gold et al^4^ with a similar signalment and imaging modality used but less favorable compared to Dyson et al^1^ in which conservative treatment in a small group of sports horses had a good outcome. In contrast to our results, a report of surgical management of radiographically evident incomplete fractures was associated with significantly better prognosis compared to conservative management, with 100% (5/5) of horses treated with internal fixation being sound at the time of the follow‐up.^7^ Horses with short incomplete fractures may represent a different manifestation of the bone injury described in our study, or the injury could be a bone injury with different pathogenesis.

If the 3 horses that had a delayed surgical intervention are excluded from our study, outcome following surgical treatment appears more promising than conservative management.

According to our study, subchondral bone injury at the level of the proximal sagittal groove of the PP occurs in horses competing in different disciplines and at different levels. The population in this study had an overrepresentation of dressage and eventing horses, which was similar to that reported by Dyson et al^1^ but dissimilar to other studies in which over 50% of the horses with diagnoses of subchondral bone damage or fracture of the sagittal groove of the PP were show jumpers.^4^, ^5^ The different populations represented likely relate to the local horse population presented to the different equine hospitals. A common factor among the different equestrian disciplines represented is the potential for intensive training of repeated movements.^1^, ^2^ Horses experiencing repetitive and likely higher loading of the distal limb may be at increased risk of developing subchondral bone injury.^14^ Most of the horses included in our study were competing at high (10/21) and intermediate (4/21) levels; however, because of the low number of cases and the lack of controls, it is not possible to establish a statistical correlation between level of exercise and occurrence of the pathology.

Traditionally, etiology of sagittal fracture of PP has been attributed to a monotonic event;^12^ however, recently, repetitive trauma to the region has also been suggested as a contributory factor.^1^, ^2^, ^15^ Authors have reported that horses with high water signal in the sagittal groove of the PP have mainly a chronic history of lameness (5/8 horses)^1^; however, in this study, acute onset of lameness occurred in 14 of 21 horses. The difference in onset may reflect more rapid presentation and diagnosis of this injury in our population of horses, or it could represent different aetiologies.^4^

Detection of sagittal groove injuries of the PP represents a clinical, diagnostic challenge.^4^ This study confirmed the previous finding that lameness in horses with subchondral bone injury of the sagittal groove of the PP is often abolished by “foot blocks,” with the majority of horses responding completely to perineural analgesia either of the palmar/plantar digital nerves performed at the level of the ungular cartilages or of the palmar/plantar nerves performed at the base of the proximal sesamoid bones.^5^, ^16^, ^17^ Proximal diffusion of local anesthetic solution from palmar digital anesthesia potentially explains how anesthesia of the palmar digital nerves at the level of the ungular cartilages may alleviate pain associated with the fetlock area.^17^, ^18^ Injury to the sagittal groove should be considered in lame horses that respond to palmar digital analgesia with no important diagnostic imaging abnormalities noted on the foot and pastern regions.^4^ Ten of the 21 horses included in the study were initially referred for foot MRI; however, when MR images were unremarkable, the decision was then made to perform fetlock MRI.

Distribution of the high water signal in our study was similar to previous reports.^1^, ^4^ When it was detected, the fracture line was within the high water signal and was mainly in the central region of the sagittal groove (4/6 horses). In our study, the dorsopalmar/plantar position of the fracture line partially mirrored the position reported by Brunisholz et al^5^ with CT, with the fractures located much more dorsally in forelimbs compared to hind limbs.^5^ The fracture described by Brunisholz and colleagues with CT likely represents the end‐stage of the osseous trauma described in this study.^5^ Arendt and Griffiths^19^ described a grading system (0‐4) for human stress fracture that was based on radiographic, scintigraphic, and MRI findings. According to this grading system, the earliest detectable imaging abnormality (grade 1) is hyperintense signal on STIR MRI sequences, indicating increased water content within the tissue. Presence of a fracture line, detected with any imaging modality, was considered the end‐stage (grade 4). Many studies seeking to improve diagnostic accuracy and efficacy of stress‐related injuries in human athletes have compared the ability of MRI and CT to detect stress reaction and stress fractures.^19^, ^20^, ^21^, ^22^, ^23^ In human medicine, high‐field MRI is considered the test of choice for early diagnosis of bone stress injuries.^20^ Increased water content within the bone, which is considered the earliest macroscopic change associated with stress injury, is visible on MRI within approximately 1 to 3 days of onset of pain.^21^ In contrast, CT is largely unable to detect bone turnover or changes in bone water content, which limits its utility for early diagnosis of stress fractures.^21^ However, when a fracture is present, the ability of CT to image the osseous anatomy and fracture configuration is superior to MRI.^22^, ^23^ The horses included in this study were probably presented at different stages of the disease, and this may account for the variable response to treatment.

In this study the horses that were managed surgically underwent placement of a cortical screw in lag fashion under radiographic guidance. The surgical techniques of subchondral perforation with a cortical bone screw through injured subchondral bone was described for horses diagnosed with subchondral bone injury of the lateral condyle of the third metatarsal bone (MTIII).^8^ Ross^8^ reported that 100% (3/3) of racehorses treated surgically in this manner raced after surgery.

In our population, the rationale for placing a screw across the area of abnormal proximal sagittal groove of the PP may vary according to the stage of the condition. At an early stage, screw placement could promote healing through compression along the damaged area and preventing further microcrack propagation, providing immediate decompression of painful, sclerotic subchondral bone and allowing marrow components from the trabecular bone access to heal the damaged subchondral bone.^8^ In the presence of a fracture line, the screw placed in lag fashion reduces the fracture line, promoting primary bone healing and preventing further propagation.

The limitations of the study include its retrospective nature and the low number of cases, leading to poor statistical power. The stringent inclusion criteria, which excluded cases with clear radiographic evidence of a fracture line in the proximal sagittal groove of PP, further limited the sample. Conservative vs surgical treatment was not randomly assigned. Moreover 3 of 9 horses treated surgically were treated conservatively in the first instance, delaying surgical intervention and artificially enhancing the success of the conservative group. Also, the fact that the only 2 imaging modalities used in the study were radiography and low‐field MRI may be a limitation. As previously discussed, MRI is the imaging modality of choice for detection of early stress‐related changes within the bone, but CT is superior in defining fracture configuration.^20^, ^23^ It is possible that low‐field MRI did not detect all the incomplete PP fractures, and this could have negatively affected the surgically planning. In addition, low‐field MRI does not detect articular cartilage abnormalities. There is the possibility that concurrent undiagnosed cartilage injury of the fetlock joint could have contributed to the poor outcome of 8 of 17 horses in our study.

In conclusion, this study compared conservative and surgical management, without an encouraging outcome for either treatment modality. Overall treatment was successful in approximately half of the horses (9 of 17), with no significant difference in outcome between horses treated conservatively vs surgically. This study provides additional clinical and diagnostic imaging information related to osseous trauma of the proximal aspect of PP in sports horses by detailing the location of MRI signal abnormalities and the similar outcome following 2 different possible treatment modalities. Horses affected by this condition seem to have a guarded prognosis for return to athletic activity. Currently, MRI is a reliable imaging modality for early detection of abnormalities within the sagittal groove of the PP; however, a better understanding of the etiology and pathogenesis of the osseous trauma of this area is required to determine the best treatment modality.

## CONFLICT OF INTEREST

The authors declare no conflict of interest related to this report.

## Supporting information

Supporting Information
