# Supplementary material for: Conservative versus surgical treatment of 21 sports horses with osseous trauma in the proximal phalangeal sagittal groove diagnosed by low‐field MRI
Source: Vet Surg. 2018 Sep 14;47(7):908–15. doi: 10.1111/vsu.12936 (PMC6690071; doi:10.1111/vsu.12936)
Supplement: Supplementary file 1 — Supporting Information [file VSU-47-908-s001.docx]

**Telephone Interview Schedule**

1. Introduction:

xx will introduce herself/himself as a veterinary surgeon from the xx. Then she/he will indicate the purpose of the call: to collect data for a research project on bone injury and incomplete fractures of the proximal phalanx (long pastern bone) in sport horses, for which their horse was previously diagnosed and treated.

1. The participant will then be read the following paragraph: “Osseous trauma of the first phalanx (bone also known as “the long pastern bone”) in sport horses is a condition that has gained increasing attention over the last few years. Only a small amount of scientific data is available about this condition. We know some things about this condition such as the clinical findings, that are not easy to see on X-rays and that MRI is probably the best way to detect the problem. If not correctly managed this type of injury can become a complete pastern fracture, which could be life-threatening to the horse. It is currently unclear what is the best treatment option when early changes of the pastern bone are detected with MRI. Some clinicians advise a conservative treatment (controlled exercise with other medical therapy) while other clinicians advise a surgical treatment (screw placed across the fracture line). Therefore we are undertaking a study with the aim of comparing the outcome between conservative and surgical management of horses diagnosed an incomplete pastern fracture using standing MRI. This study, which looks back retrospectively at clinical cases, should provide valuable information for veterinary surgeons when attending sport horses with a suspected incomplete fracture of the long pastern bone”.
2. Your participation in this study is voluntary and you do not need to provide the information we request. In addition, you can choose to withdraw your horse’s information from the study at any time.
3. Your data is anonymised and no personal or horse details are required, kept or published. We are simply trying to find out how your horse has progressed following treatment for this condition. It will not be possible to identify owner or horse from publication.
4. If you are at all concerned about the information we have requested or you would like to speak to the study leader, then please contact xx (contact details are provided at the end of this document and will be given to the client)
5. Are you happy to proceed with the interview? Yes ☐ No ☐
6. Is your horse (mention name) still alive? Yes ☐ No ☐
7. If not, I am really sorry to hear about that. Was the horse put down because of the condition or because of an unrelated issue? Yes ☐ No ☐
8. When was your horse last examined by a veterinary surgeon for the same condition? ________________________
9. Is your horse currently lame? Yes ☐ No ☐
10. Is the horse lame at the walk? Yes ☐ No ☐
11. Is the horse lame at the trot? Yes ☐ No ☐
12. Can you grade the lameness? Mild ☐ Moderate☐ Severe☐ I don’t Know☐
13. Is the lameness related to the previous long pastern bone condition?

Yes ☐ No ☐ I don’t Know☐

1. Can you see any blemish on the horse previously affected limb?

Yes ☐ No ☐ I don’t Know☐

1. If yes, which kind of blemish you noticed?

- Fetlock effusion ☐
- Bone reaction over the surgical site☐
- Bone reaction on the front of the pastern ☐
- Others _____________________________

1. What is your horse’s present level of activity? (Pasture☐ Handwalking☐ Light ridden exercise☐ Training for competition☐ Competing☐)
2. Is the horse currently performing at the previous level? Yes ☐ No ☐
3. If not, is he performing at higher or lower level? ­­________________________________________
4. Thank for participation in the study
